# Medical Reform

**Published:** 1842-07

**Authors:** 


### Medical Reform.

It being now generally understood that Sir James Graham's bill for remodelling some parts of the profession is likely to be *introduced* into Parliament this session, we earnestly call on all our readers—especially on those of the class of general practitioners—to be early on the watch to learn and to study its provisions. As it cannot *pass* this session, there will be time for examining and, if need be, for resisting it. In the meantime, we also strongly recommend to their notice the scheme of rational and practical reform, proposed in an admirable pamphlet just published by Sir James Clark.* Unless as much is granted to *the great body of the profession*—that is, to the general practitioners—as is contended for in this pamphlet, the reform of the corporations, said to be contemplated by the bill, will be, to say the least, utterly nugatory as a means of healing existing discontents and discords, and of raising the profession, generally, in the estimation of the public and their own.

* Remarks on Medical Reform, in a Letter addressed to Sir James Graham, Bart., &c. &c. By Sir Jas. Clark, Bart., M.D. F.R.S., Physician to the Queen, &c.—Lond. Murray.

